# Placental Transfer of Persistent Organic Pollutants: A Preliminary Study on Mother-Newborn Pairs

**DOI:** 10.3390/ijerph10020699

**Published:** 2013-02-07

**Authors:** Maria Grazia Porpora, Renato Lucchini, Annalisa Abballe, Anna Maria Ingelido, Silvia Valentini, Eliana Fuggetta, Veronica Cardi, Adele Ticino, Valentina Marra, Anna Rita Fulgenzi, Elena De Felip

**Affiliations:** 1 Department of Gynaecology, Obstetrics and Urology, “Sapienza” University of Rome, Policlinico Umberto I, Viale del Policlinico 155, 00161 Rome, Italy; E-Mails: mariagrazia.porpora@uniroma1.it (M.G.P.); eliana.fuggetta@gmail.com (E.F.); veronica.cardi@gmail.com (V.C.); adele.ticino@yahoo.com (A.T.); 2 Perinatology and Childcare, “Sapienza” University Policlinico Umberto I, Viale del Policlinico 155, 00161 Rome, Italy; E-Mail: renato.lucchini@uniroma1.it; 3 Toxicological Chemistry Unit, Department of the Environment and Primary Prevention, Istituto Superiore di Sanità, Viale Regina Elena 299, 00161 Rome, Italy; E-Mails: annalisa.abballe@iss.it (A.A.); annamaria.ingelido@iss.it (A.M.I.); silvia.valentini@iss.it (S.V.); valentina.marra@iss.it (V.M.); annarita.fulgenzi@iss.it (A.R.F.); elena.defelip@iss.it (E.D.F.)

**Keywords:** *p,p’*-DDE, β-HCH, HCB, polychlorinated biphenyls (PCBs), perfluorooctane sulfonate (PFOS), perfluorooctanoic acid (PFOA), *in utero* exposure, placental transfer

## Abstract

The aim of this study was to characterize the placental transfer of some environmental pollutants, and to explore the possibility of quantitatively predicting *in utero* exposure to these contaminants from concentrations assessed in maternal blood. Levels of toxic substances such as pesticides (*p,p’*-DDE, β-HCH, and HCB), polychlorinated biphenyls (PCBs), perfluorooctane sulfonate (PFOS), and perfluorooctanoic acid (PFOA) were determined in serum samples of 38 pregnant women living in Rome and in samples of cord blood from their respective newborns. The study was carried out in the years 2008–2009. PCB mean concentrations in maternal serum and cord serum ranged from 0.058 to 0.30, and from 0.018 to 0.064 ng/g·fw respectively. Arithmetic means of PFOS and PFOA concentrations in mothers and newborns were 3.2 and 1.4 ng/g·fw, and 2.9 and 1.6 ng/g·fw. A strong correlation was observed between concentrations in the maternal and the foetal compartment for PFOS (Spearman r = 0.74, p < 0.001), PFOA (Spearman r = 0.70, p < 0.001), PCB 153 (Spearman r = 0.60, p < 0.001), HCB (Spearman r = 0.68, p < 0.001), PCB 180 (Spearman r = 0.55, p = 0.0012), and *p,p’*-DDE (Spearman r = 0.53, p = 0.0099). A weak correlation (p < 0.1) was observed for PCBs 118 and 138.

## 1. Introduction

Persistent organic pollutants (POPs) are a group of toxic chemicals widely distributed in the environment which includes polychlorinated dibenzodioxins (PCDDs), polychlorinated dibenzofurans (PCDFs), polychlorinated biphenyls (PCBs), organochlorinated pesticides and perfluorinated organic compounds (PFCs). These chemicals have been recognised as a threat to the environment and human health because of their high chemico-physical stability, long environmental and biological persistence, and a wide range of toxic effects. A number of studies suggest that exposure to PCBs and organochlorinated pesticides may lead to increased cancer risk [1,2,3,4,5], nervous system damage [6,7], reproductive disorders [8,9,10,11], and immune system disruption [12,13] .

As to the two most abundant members of the perfluorinated compound family, perfluorooctane sulfonate (PFOS) and perfluorooctanoic acid (PFOA), the main effects observed in animal models are hepatotoxicity, developmental toxicity, immunotoxicity and hormonal effects. Animal studies also suggest that at relatively high doses PFOS and PFOA may be carcinogenic [14,15,16,17,18,19].

As a consequence of POP toxic effects on human health, a number of regulatory measures have been undertaken at an international level to eliminate or reduce their release into the environment and human exposure. Human exposure monitoring over time allows one to evaluate if measures undertaken are effective in reducing the release of POPs into the environment. Biomonitoring is recognised as the most effective tool to characterize exposure to POPs since it provides the direct measurement of the internal dose of a chemical resulting from all sources and pathways, which represents the most appropriate dose-metric for risk assessment [20]. 

Exposure of infants and children is one of the major points of concern associated to POPs. In fact, many epidemiological studies suggest that prenatal and postnatal exposure to organochlorinated compounds is linked to a number of adverse effects in children such as neurodevelopmental delays and disorders [21,22,23,24,25]. In addition, effects that may become evident later in life [13] are also associated with exposure that occurs in this stage of life. With regard to PFCs, prenatal exposure to PFOS and PFOA has been associated to decreased fecundity and reduced sperm counts, motility and morphology [26,27], although conflicting results have been reported by different studies [17,26,28,29]. Elevated exposures to PFCs in children aged 5 and 7 years have been associated with a decreased immune response to childhood vaccines, which might reflect a more general immune system deficit [30].

While perinatal exposure to POPs through breastfeeding may be quite well characterized through the analysis of breast milk and the application of appropriate toxicokinetic models, the characterization of *in utero* exposure through the analysis of POPs in cord serum is still inadequate. In fact, practical and ethical problems often hamper the availability of cord serum samples and/or of the sample volume needed for the analysis of these lipophilic compounds in a matrix, such as cord blood, characterized by a low fat content. 

The main aim of the present human biomonitoring study was to assess if a quantitative relationship may be established to characterize the placental transfer of a group of POPs present in greatest abundance in human tissues, and therefore to predict *in utero* exposure to these contaminants from levels assessed in maternal blood.

The most abundant PCB congeners (the so-called “indicator” PCBs 28, 52, 101, 138, 153, and 180 plus PCBs 118 and 156), the organochlorinated pesticides *p,p'*-dichlorodiphenyldichloroethylene (*p,p’*-DDE), β-hexachlorocyclohexane (β-HCH) and hexachlorobenzene (HCB), and the two main members of the family of the perfluorinated compounds (PFOS and PFOA) were therefore analysed in matched mother-newborn pairs.

## 2. Materials and Methods

### 2.1. Recruitment of Study Participants, Sample Collection and Analysis

Women subject enrolment was carried out between May 2008 and May 2009 at the Department of Gynaecology-Obstetrics and Urology, Policlinico Umberto I, University of Rome “Sapienza”. The study, approved by the Local Research Ethic committee, involved 38 mother-child pairs. 

A sample of about 30 mL of blood was withdrawn from each woman at the time of hospitalization or the next hours after delivery. Cord blood samples were taken during the delivery, either vaginal or by caesarean section, between childbirth and placental expulsion. All women gave informed consent for themselves and for their infants before participating in the study. 

Blood samples were centrifuged to obtain serum. Only a few milliliters of serum were obtained from the umbilical cord blood, due to the high hematocrit (average value of 44–62%) of fetal blood [31]. Serum samples were stored at −20 °C until time of analysis. 

Birth weight of infants was measured within 1 hour from delivery with an electronic balance and recorded to the nearest gram. Birth crown-heel length and head circumference were measured within 1 h with a Harpenden neonatometer and an inelastic tape, respectively, and recorded to the nearest millimeter. Percentile was calculated using Italian Neonatal Anthropometric Charts [32]. Apgar score was evaluated by the attending neonatologist in the delivery room at one and five minutes after birth.

### 2.2. Analysis

#### 2.2.1. Organochlorinated Pesticides and PCBs

An aliquot of about 4–12 mL of each maternal serum sample and about 3–5 mL of each cord serum were added with a mixture of ^13^C labelled PCBs (28, 52, 101, 118, 138, 153, 156, 180), and a mixture of ^13^C labelled pesticides (*p,p’*DDE, HCB, β−HCH), and allowed to rest overnight at 4 °C. Formic acid/2-propanol (4/1, v/v, 15 mL) were added to the samples, which were sonicated and extracted by manual shaking with *n*-hexane. After centrifugation, the organic phase was removed and collected. This extraction process was repeated two times. The *n*-hexane extracts were treated with concentrated sulphuric acid, separated by centrifugation and then concentrated and transferred into 1 mL autosampler vials. After addition of 1 μL of tetradecane, extracts were concentrated to dryness, an isooctane solution of the injection standard (200 μL) was added, and samples were quantified [33,34,35]. 

Instrumental analysis was carried out by ion trap mass spectrometry (Thermo Finnigan Polaris Q) coupled to high resolution gas chromatography used in the MS–MS mode. The isotope dilution technique was applied throughout. Recoveries ranged from 60–120%.

Analytical reliability was warranted by the use of an in-house validated method [33]. The laboratory has considerable experience in the analysis of halogenated organic microcontaminants and periodically participates in interlaboratory comparison exercises and proficiency tests on the analysis of PCDDs, PCDFs, PCBs, organochlorinated pesticides, and brominated flame retardants in dietary, biological, and environmental matrices.

#### 2.2.2. PFOS and PFOA

An aliquot of about 250 μL of each serum sample was fortified with a mixture of ^13^C-labelled PFOS and PFOA and allowed to stand overnight at 4 °C. Extraction was performed with acetonitrile by manual shaking in a centrifuge tube, followed by centrifugation at 3,500 revolutions per minute (rpm) for 10 min. Acetonitrile aliquots were removed, collected in centrifuge tubes, carefully concentrated by a multiple samples evaporator system and transferred to an autosampler vial to undergo instrumental analysis [36]. Instrumental analysis was carried out by HPLC (Waters 2695 separations module) interfaced to a mass spectrometer (Waters Micromass Quattro micro API) operated in the electrospray negative mode. Data were acquired using multiple reaction monitoring (MRM). The isotope dilution technique was applied throughout. Recovery ranges of ^13^C-labelled internal standards were 70–110%. Analysis of blanks and control samples was systematically carried out to check the analytical reliability. Limits of detection for PFOS and PFOA were 0.05 ng/g·fw and 0.1 ng/g·fw, respectively [36].

### 2.3. Statistical Analysis

The Shapiro-Wilk test was used to test the normal distribution of data. The Spearman test was used to evaluate the correlation between concentrations of organochlorinated pesticides, PCBs and PFCs in maternal and cord serum and the correlation between concentrations of all the analytes. Linear regression analysis was used to investigate the transfer behaviour of all compounds. The Spearman test was also used to evaluate the correlation between levels of POPs in maternal and foetal serum and gestational age, Apgar scores and weight at birth. All statistical analyses were carried out using STATISTICA, version 8.0 (StatSoft, Inc., Tulsa, Oklahoma). 

## 3. Results

A total of 38 Italian Caucasian women aged 26–45 years (mean age, 34.5 years) and their newborns were included in this study. In Table 1, the general characteristics of the women and their infants are reported. Out of the enrolled women, 23 women were at their first pregnancy. Mean gestational age was 39 weeks (range 35–42 weeks).

**Table 1:** General characteristics of the enrolled women and their newborns.

Characteristics of Women (*n = 38*)	Min	Median	Mean	Max
Age	26.0	34.0	34.6	45.0
Gestational age (weeks)	35	39	39	42
BMI pre pregnancy	18.0	22.7	22.3	25.2
Characteristics of newborns (*n* *=* *38*)				
Baby birth weight (g)	2,190	3,213	3,239	4,420
Baby birth length (cm)	45.1	49.0	49.6	57.0
Baby head circumference (cm)	31.0	34.5	34.1	36.0
Apgar score (1 min)	4.0	8.0	8.2	9.0
Apgar score (5 min)	7.0	9.0	9.4	10.0

Sample volume was sufficient for the analysis of PFOS and PFOA in all samples, while only 32 out of the 38 samples had a volume sufficient for the analysis of the organochlorinated compounds. With regard to maternal serum samples, 24% had concentrations below the limit of quantification (LOQ) of β-HCH and PCB 118, 16% of PCBs 180, 138 and 153, and <5% of HCB, *p,p’*-DDE, PFOS and PFOA. LOQ values ranged from 1 to 10 pg/g·fw for PCBs and organochlorinated pesticides and were of 0.1 and 0.5 ng/g·fw for PFOS and PFOA respectively.

With regard to cord serum, the percent of samples with concentrations below LOQs were 74% for β-HCH, 53% for PCB 118, 47% for HCB, 39% for *p,p’*-DDE, 29% for PCB 138 and PFOA, 26% for PCB 153, 18% for PCB 180, and 3% for PFOS. Concentrations of PCBs 28, 52, 101, and 156 were always below their respective LOQs in cord blood samples.

Concentrations of β-HCH, HCB, *p,p’*-DDE, and PCBs 118, 138, 153 and 180, PFOS and PFOA assessed in maternal and cord serum are shown in Table 2, summarized by arithmetic means, medians, minimum and maximum values, 25th and 75th percentiles. Values are expressed on a fresh weight basis since lipid content of cord serum samples could not be determined because the majority of cord samples were hemolyzed, this unabling the application of enzymatic methods currently used for lipid determination [37].

The pesticide found at the highest concentration in both maternal and cord serum samples was *p,p’*-DDE. Mean concentrations of this pollutant in maternal serum and cord serum were 2.0 and 0.52 ng/g·fw respectively. Concentrations of HCB and β-HCH in maternal serum and cord serum were 0.31 and 0.13 in cord ng/g·fw, and 0.16 ng/g·fw and 0.047 ng/g·fw respectively.

As to the PCB congeners, a similar pattern in relative abundance was observed in the maternal and foetal compartments, with levels of PCB 153 > PCB 180 > PCB 138 > PCB 118. PCB mean concentrations in maternal serum and cord serum were 0.30 and 0.064 ng/g·fw for PCB 153, 0.14 and 0.030 ng/g·fw for PCB 138, 0.22 and 0.043 ng/g·fw for PCB 180. Mean concentrations of the dioxin-like PCB 118 were 0.058 and 0.018 ng/g·fw in maternal and cord serum, respectively.

Arithmetic means of PFOS concentrations were 3.2 and 1.4 ng/g·fw in mothers and infants, respectively, while PFOA mean concentrations were 2.9 and 1.6 ng/g·fw.

**Table 2:** Serum concentrations (ng/g, fresh weight) of organochlorinated pesticides (β-HCH, HCB and DDE), four congeners of polychlorobiphenyls (PCBs) and perfluorinated compounds (PFCs) in maternal and cord samples. Values rounded off to two figures. Concentrations <LOQ included in the analysis.

Compounds	N	Min	P_25_	Median	Mean	P_75_	Max
*β-HCH* Maternal serum	32	0.0090	0.039	0.065	0.16	0.13	1.5
*β-HCH* Cord serum	32	0.0014	0.014	0.024	0.047	0.057	0.25
*HCB* Maternal serum	32	0.060	0.12	0.17	0.31	0.44	1.4
*HCB* Cord serum	32	0.003	0.020	0.044	0.13	0.11	1.2
*p,p’-DDE* Maternal serum	32	0.24	0.57	0.78	2.0	1.3	25
*p,p’-DDE* Cord serum	32	0.0050	0.12	0.22	0.52	0.48	4.0
*PCB 118* Maternal serum	32	0.0006	0.029	0.055	0.058	0.068	0.219
*PCB 118* Cord serum	32	0.0008	0.002	0.013	0.018	0.030	0.068
*PCB 138* Maternal serum	32	0.033	0.094	0.12	0.14	0.15	0.45
*PCB 138* Cord serum	32	0.0017	0.016	0.031	0.030	0.043	0.073
*PCB 153* Maternal serum	32	0.090	0.17	0.27	0.30	0.32	0.94
*PCB 153* Cord serum	32	0.023	0.037	0.057	0.064	0.084	0.16
*PCB 180* Maternal serum	32	0.040	0.14	0.20	0.22	0.25	0.61
*PCB 180* Cord serum	32	0.0052	0.027	0.041	0.043	0.051	0.11
*PFOS* Maternal serum	38	0.062	1.9	2.9	3.2	3.9	13
*PFOS* Cord serum	38	0.23	0.75	1.1	1.4	1.8	3.7
*PFOA* Maternal serum	38	0.20	1.9	2.4	2.9	4.0	9.1
*PFOA* Cord serum	38	0.17	0.29	1.6	1.6	2.2	5.0

### 3.1. Correlation Analyses

For all analytes, data distributions were approximately log-normal. The Spearman non-parametric test was applied to all compounds, followed by a linear regression analysis performed on log-transformed data.

As shown in Table 3, a strong correlation was observed between concentrations in the maternal and the foetal compartment for PFOS (Spearman r = 0.74, p << 0.001), PFOA (Spearman r = 0.70, p << 0.001), PCB 153 (Spearman r = 0.60, p < 0.001), HCB (Spearman r = 0.68, p < 0.001), PCB 180 (Spearman r = 0.55, p = 0.0012), and *p,p’*-DDE (Spearman r = 0.53, p = 0.0099). A weak correlation (p < 0.1) was observed for PCBs 118 and 138, and not significant correlation was found for β-HCH.

The regression analysis confirmed the results of the Spearman test and showed linear correlation between maternal and cord log-transformed concentrations for all the analytes with the exception of β-HCH, PCB 118 and PCB 138. With the exception of these latter compounds regression equations were calculated for all analytes. Intercepts and regression coefficients are reported in Table 3. 

**Table 3:** Results from the Spearman and Pearson correlation analysis. Pearson regression was performed on log transformed data.

Cord serum *vs.* maternal serum	β-HCH	HCB	*p,p’*-DDE	PCB 118	PCB 138	PCB 153	PCB 180	PFOA	PFOS
*N*	10	20	23	17	27	28	31	26	37
*Spearman correlation*									
Coefficient (r)	0.21	0.68***	0.53**	0.45*	0.33 *	0.60***	0.55**	0.70***	0.74***
*p*	0.56	0.00088	0.0099	0.073	0.098	0.0007	0.0012	<<0.001	<<0.001
*Pearson correlation*									
Coefficient (r)	0.27	0.69***	0.55**	0.3	0.29	0.57**	0.51**	0.75***	0.64***
*p*	0.44	0.0008	0.0064	0.24	0.14	0.0017	0.0034	<<0.001	<<0.001
Intercept	–	−0.353	-0.45	–	–	−0.881	−0.973	−0.033	−0.298
Regression coefficient	–	1.130	0.686	–	–	0.551	0.661	0.768	0.78

* p < 0.1; ** p < 0.05; *** p < 0.001.

A significative (p < 0.05) correlation was found among the concentrations of all compounds, with the exception of PFOA, which correlates only with PFOS, and of PFOS, which does not correlate with PCB 118 and *p,p’*-DDE. 

No association could be observed for levels in maternal and cord blood, with birth weight and gestational age. However, a statistically significant correlation (Spearman test, p < 0.1) was found between increasing levels of PCBs 118 (16 data) and 138 (22 data) in cord serum and decreasing Apgar score at 1 min (A1) and 5 min (A5) post-birth.

## 4. Discussion and Conclusions

Because of the importance of the fetal period with regard to development and differentiation, *in utero* exposure to POPs is of particular concern. Many of these compounds are in fact toxic for the immune, neurological, and endocrine systems, which experience critical developmental stages in the fetus. Transfer of toxic chemicals from maternal to fetal compartment across the placenta is similar to transfer across other biological membranes, and known to increase as the fetal growth rate increases [38].

This study addressed the partition of priority POPs between maternal and fetal serum, with the main objective to explore if a quantitative correlation could be defined to predict *in utero* exposure from maternal serum levels. To this aim, we enrolled a group of women from Rome, a city characterised by the absence of major industrial activities.

Concentrations of organochlorinated pollutants assessed in the group of women enrolled in the present study, expressed on a lipid basis for comparative purposes, are in agreement with those found in groups of women of the same age, determined by our group in studies conducted in the same years [39]. Levels of PFCs in maternal and cord serum observed in this study are generally lower than those found in other countries in the years 2004–2010 [26,28,40,41,42,43], and in agreement with levels detected in Germany and South Africa in the same period [44,45]. Our data confirm a comparatively low exposure of the Italian general population to PFCs, as we had already observed in a previous study [36].

The correlation analysis of measured POP concentrations shows that samples with comparatively higher levels of PCBs also have higher concentrations of organochlorinated pesticides, but not necessarily of PFOS and PFOA. This finding is not surprising because exposure to PCBs and organochlorinated pesticides occurs through the same routes (mainly diet, and primarily consumption of food of animal origin with high fat content), while exposure to PFOS and PFOA occurs via different routes. In fact, although food is considered the major intake pathway of PFCs in humans, release from food packaging products and inhalation of contaminated house dust may contribute a non-negligible fraction to the overall exposure [19].

As to the inverse correlation we observed between Apgar scores A1 and A5 and levels of PCB 118, this result is in line with what was observed by some authors in similar studies [50,51]. Nevertheless, because of the small number of data available for analysis, this finding can only be considered weakly indicative and therefore needs to be further investigated with a larger dataset.

Most of the POPs analysed were shown to cross the placental barrier, since they could be found in the fetal compartment, although the frequency of detection varied, ranging from 27% for β-HCH to nearly 100% for PFOS and PCB 180, and to 100% of PFOA. Because all the analysed chemicals share common physical-chemical properties, it is very likely that, also for undetected compounds, a placental passage may occur, and they were not detected in cord serum samples only because of the small amount of cord sample (< 5 mL) available. Analysis of the concentration ratios between the fetal and maternal compartments (Table 4) shows that the placental barrier partially only reduces the transport to the fetus and that some compounds, such as PFOA, and PCB 118 (and, to a lesser extent, PFOS, PCB 138 and *p,p’*-DDE) may even concentrate in the fetal compartment. 

**Table 4:** POP ratios (%) between concentrations in fetal and maternal compartments.

Compounds	N	Min	Mean	Max
β-HCH	10	2	18	54
HCB	20	5	39	100
*p,p’*-DDE	23	5	40	106
PCB 118	17	18	63	130
PCB 138	27	6	33	114
PCB 153	28	13	27	74
PCB 180	31	4	21	40
PFOS	37	10	46	110
PFOA	26	17	87	177

Although a straightforward comparison with results from other similar studies is hindered by a number of factors (such as the way to express concentrations, and the statistical approach) our results are consistent with findings obtained in another study carried out in Italy, in the polluted urban area of Brescia [46], which evidenced a linear correlation between levels of *p,p’*-DDE, HCB, and total PCBs in maternal and cord serum, and with a study recently published by Needham *et al.* [47]. As to PFCs, our results confirm the good correlation between the maternal and the foetal compartment consistently observed for PFOS in published studies [44,47,48,49], while studies carried out on PFOA provide, on the whole, conflicting results [44,47,48,49].

The results of this study, to be considered preliminary because of the small number of samples analysed, show that, for a number of priority POPs (PFOS, PFOA, PCB 153, PCB 180, HCB and *p,p’*-DDE), the placental transfer may be estimated through regression equations from concentrations assessed in maternal blood. A further investigation on a larger number of mother-newborn pairs would allow to better describe the quantitative relationships which characterize the transfer behaviour of priority POPs to the fetal compartment.
